# Layer-by-Layer Biopolymer Assembly for the *In Situ* Fabrication of AuNP Plasmonic Paper—A SERS Substrate for Food Adulteration Detection

**DOI:** 10.1021/acsomega.3c05966

**Published:** 2024-02-20

**Authors:** Nopparat Viriyakitpattana, Chanoknan Rattanabut, Chutiparn Lertvachirapaiboon, Dechnarong Pimalai, Suwussa Bamrungsap

**Affiliations:** † National Nanotechnology Center, National Science and Technology Development Agency, Thailand Science Park, Phahonyothin Road, Khlong Nueng, Khlong Luang, Pathum Thani 12120, Thailand; ‡ Thai Packaging Centre, Thailand Institute of Scientific and Technological Research, Phahonyothin Road, Chatuchak, Bangkok 10900, Thailand

## Abstract

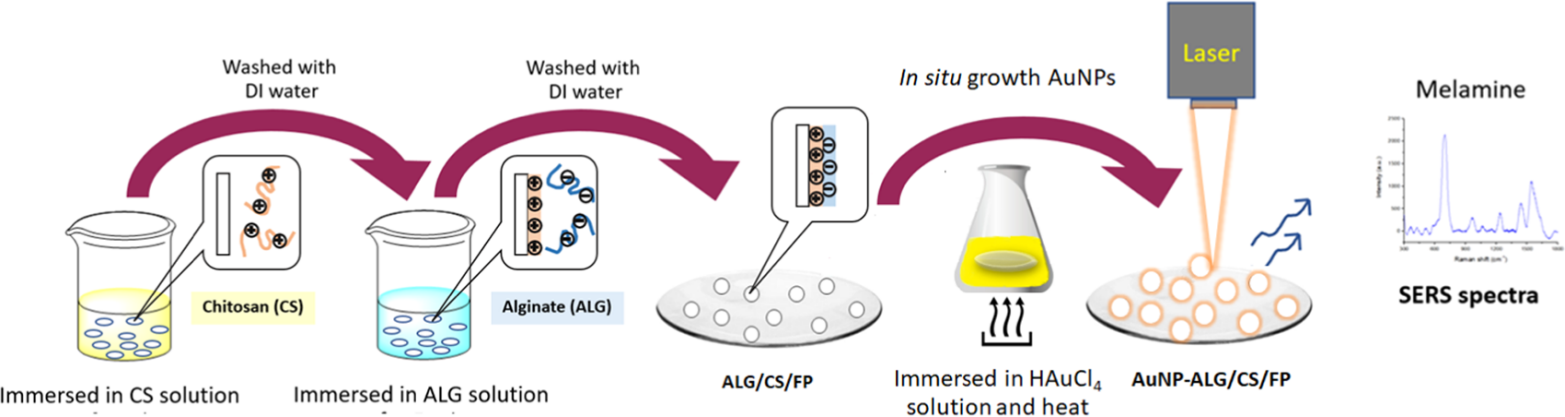

Here, we introduce an environmentally friendly approach to fabricate a simple and cost-effective plasmonic paper for detecting food additives using surface-enhanced Raman spectroscopy (SERS). The plasmonic paper is fabricated by *in situ* growth of gold nanoparticles (AuNPs) on filter paper (FP). To facilitate this green fabrication process, we applied a double-layered coating of biopolymers, chitosan (CS) and alginate (ALG), onto the FP using a layer-by-layer (LbL) assembly through electrostatic interactions. Compared to single-layer biopolymer coatings, double-layered biopolymer-coated paper, ALG/CS/FP, significantly improves the reduction properties. Consequently, effective *in situ* growth of AuNPs can be achieved as seen in high density of AuNP formation on the substrate. The resulting plasmonic paper provides high SERS performance with an enhancement factor (EF) of 5.7 × 10^10^ and a low limit of detection (LOD) as low as 1.37 × 10^–12^ M 4-mercaptobenzoic acid (4-MBA). Furthermore, it exhibits spot-to-spot reproducibility with a relative standard deviation (RSD) of 8.2% for SERS analysis and long-term stability over 50 days. This paper-based SERS substrate is applied for melamine (MEL) detection with a low detection limit of 0.2 ppb, which is sufficient for monitoring MEL contamination in milk based on food regulations. Additionally, we demonstrate a simultaneous detection of β-agonists, including ractopamine (RAC) and salbutamol (SAL), exhibiting the multiplexing capability and versatility of the plasmonic paper in food contaminant analysis. The development of this simple plasmonic paper through the LbL biopolymer assembly not only paves the way for novel SERS substrate fabrication but also broadens the application of SERS technology in food contaminant monitoring.

## 1 Introduction

Over the past few decades, food safety has drawn intensive attention and become a growing concern. The occurrence of various food contaminants, such as foodborne pathogens, pesticides, food additives, and toxins, can lead to immediate health issues and contribute to long-term effects relevant to hundreds of diseases or sometimes lethal consequences.^1^ Most of the food safety analysis uses conventional methods, which are chromatographic-based “wet chemistry” techniques, such as gas chromatography (GC) and high-performance liquid chromatography (HPLC), followed by mass spectroscopy (MS).^2−4^ However, these methods still have limitations in high costs and difficulties in sample preparation before analysis, including the need of an internal standard. Therefore, detection platforms that are simple, highly sensitive, cost-effective, and suitable for onsite operation or are able to be operated in a small laboratory are much needed.

Surface-enhanced Raman spectroscopy (SERS) is one of the powerful techniques with notable advantages, such as high sensitivity, rapid analysis, simple operation, label-free approach, and quantitative analysis.^5−7^ SERS provides molecular fingerprints derived from the frequency shifts correlated to their molecular vibrations.^8,9^ Due to its benefits, SERS has found applications in a broad spectrum, including biomedical sensors,^10,11^ chemical sensors,^12^ and material characterization.^13,14^ Generally, SERS requires the localization or close adsorption of target analytes to substrates, which typically comprise noble metal nanostructures, such as gold, silver, and copper. Several SERS substrates have been fabricated by depositing metallic nanostructures or nanoparticles onto surfaces, such as glass slides, silicon wafers, and aluminum films. However, many of these SERS substrate fabrication methods still have drawbacks, including being expensive, time-consuming, complicated, and requiring sophisticated processes.

Recently, plasmonic papers have emerged as effective SERS substrates because of their cost-effectiveness, lightweight, flexibility, portability, and biodegradability.^15,16^ When utilizing SERS on these substrates, a liquid sample can be simply loaded *via* capillary force by dipping the plasmonic paper into the sample. Then, paper can act as a microfluidic for liquid sample transportation and the analytes can be concentrated at a specific area by solvent evaporation, resulting in an intense SERS signal.^17−19^ To fabricate a plasmonic paper, metal nanoparticles are typically synthesized in a colloidal phase and subsequently deposited onto the paper using various procedures, such as dipping,^20^ vacuum filtration,^21^ inkjet printing,^22^ and physical vapor deposition.^23^ However, the drawbacks of these methods are the ability to control uniformity, density, and ineffective adhesion of the plasmonic nanoparticles on the substrates, which directly affects SERS performance.

To address these challenges, the *in situ* growth of plasmonic nanoparticles directly on paper substrates has been explored. This approach enables the formation of metal nanoparticles in a densely uniform and controllable manner on the fibrous structure of cellulose, resulting in excellent SERS performance and high reproducibility.^24−26^ For instance, Cheng et al. fabricated a plasmonic paper by *in situ* growth of silver nanoparticles (AgNPs) on filter paper (FP) through a silver mirror reaction, leading to a highly uniform and controlled structure.^24^ Similarly, Kim and co-workers suggested a paper-based SERS platform *viain situ* growth of AgNPs known as the successive ionic layer absorption and reaction (SILAR) method using NaBH_4_ as a reductant.^25^ This approach yielded a paper-based SERS substrate with an excellent SERS enhancement property and uniform AgNP formation. In previous studies, the *in situ* growth of metal nanoparticles on paper often used conventional reducing agents, such as sodium borohydride, citrate, *etc*. However, the excess reagents and byproducts from typical reducing reagents can be potentially toxic to the environment and possibly cause biological risks.^27^ Hence, employing a green and ecofriendly reduction method is considered as an alternative and attractive approach for the *in situ* synthesis of metal nanoparticles on paper to provide plasmonic-based SERS substrates.

Polysaccharides are favorable as both green reducing and stabilizing agents for metal nanoparticle synthesis due to their remarkable reduction properties, excellent stability, and cost-effectiveness. Chitosan (CS), an *N*-deacetylated derivative of chitin and one of the most abundant natural biodegradable polysaccharides, has been reported to effectively reduce gold salt to zerovalent AuNPs without the need of additional reducing agents. Additionally, CS acts as a stabilizing agent for AuNPs, exhibiting its dual functionality at the same time.^28−30^ Apart from CS, alginate (ALG), a negatively charged polysaccharide, has also been explored for green synthesis of AuNPs.^31^ ALG is a polysaccharide derived from copolymerization of α-l-guluronic acid and β-d-mannuronic acid containing numerous carboxyl and hydroxyl groups along the backbone. During the synthesis, CS and ALG facilitate the reduction of Au(III) ions to Au(0) through charge transfer between empty d-orbitals in Au atoms and lone-pair electrons in N or O atoms of polar functional groups, including amino, carboxyl, or hydroxyl groups, resulting in the formation of AuNPs.^30,31^ Meanwhile, those polar functional groups possessing either positive or negative surface charges can prevent AuNPs from aggregation and offer highly dispersive AuNPs. To date, the combination of CS and ALG as green reducing agents for plasmonic paper fabrication remains unexplored. It is expected that the bilayer of CS and ALG can enhance the amount and density of AuNP formation on the paper, thereby improving the SERS performance of the resulting substrate.

Herein, we present the *in situ* synthesis of AuNPs on FP using a green reduction approach, aiming to fabricate a paper-based SERS substrate for ultrasensitive detection of food adulteration. To achieve this, we employed a layer-by-layer (LbL) assembly technique to generate double layers of CS and ALG on FP (ALG/CS/FP). This assembly was facilitated by electrostatic interactions due to the presence of rich amine contents in CS and the abundance of carboxyl groups in ALG. Subsequently, AuNPs were grown *in situ* on the ALG/CS/FP substrate through the reduction of gold chloride by the ALG/CS double layer, resulting in plasmonic paper. For comparison, we utilized single layers of CS- and ALG-coated FPs (CS/FP and ALG/FP) for the *in situ* growth of AuNPs on FP. After optimization and characterization, the SERS performance of the plasmonic paper was further evaluated, including the EF, sensitivity, uniformity, and stability using 4-MBA as a Raman probe. Notably, the fabricated plasmonic paper demonstrated excellent sensitivity in the quantitative detection of melamine (MEL), achieving a low detection limit of 0.2 ppb. Moreover, we demonstrated the versatility of the developed paper-based SERS platform through multiplex detection of β-agonists, including ractopamine (RAC) and salbutamol (SAL), highlighting its broad applicability in food analysis.

## 2 Results and Discussion

### 2.1 Fabrication and Characterization of the Plasmonic Paper

**Scheme 1:**
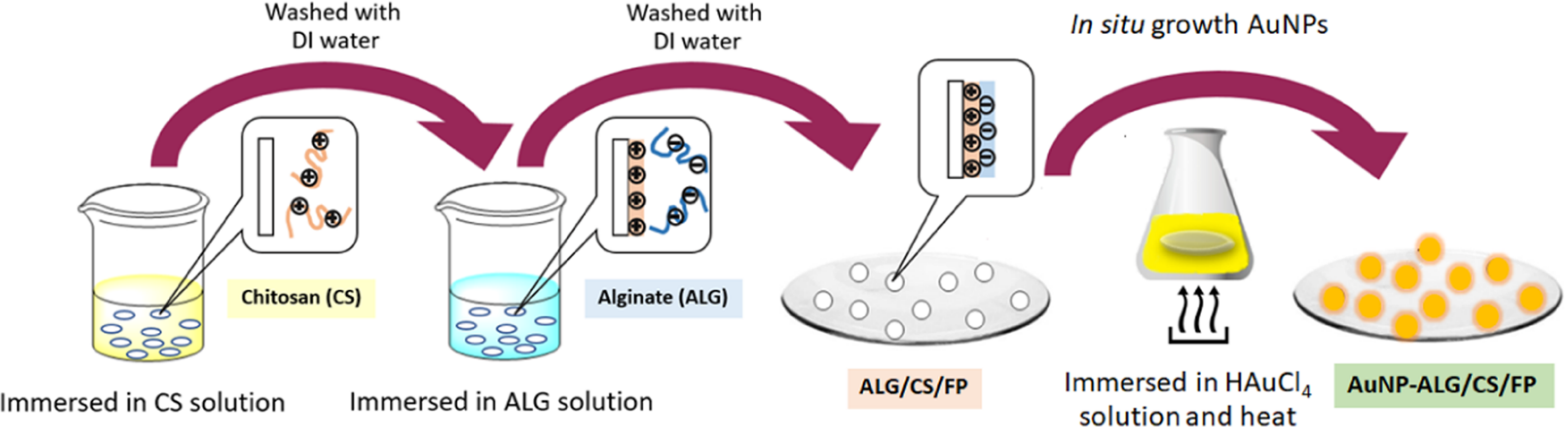
Schematic Illustration Represents Each Step of Plasmonic Paper Fabrication

In this study, a laboratory-grade FP was employed as a substrate for the fabrication of plasmonic paper due to its good absorption, high surface area, simplicity, and cost-effectiveness. The *in situ* synthesis of AuNPs was performed to tightly deposit the nanoparticles with high density and homogeneity on the FP. Instead of using conventional reducing agents, two biopolymers, CS and ALG, were applied for the green reduction of AuNPs on the FP in order to reduce wastes and byproducts from the synthesis, which might be toxic to the environment and health. As demonstrated in Scheme 1, CS was precoated on the FP, followed by ALG as reducing agents through the LBL assembly employing electrostatic interactions. The resultant ALG/CS/FP was then immersed in a HAuCl_4_ solution and heated to facilitate AuNP formation, leading to the fabrication of the plasmonic paper denoted as AuNP-ALG/CS/FP.

To validate the deposition of ALG/CS double layers on the FP through the LbL assembly, we determined the thickness of the precoated FPs by cross-sectional scanning electron microscopy (SEM), as shown in Figure S1. The thickness of the bare FP increased from 127.23 μm after monolayer coating of FP with CS and ALG to 136.36 and 145.45 μm, respectively, whereas the thickness of ALG/CS/FP double-layered coating was increased up to 157.73 μm. It is obvious that the increase in the thickness of the paper is related to the number of biopolymer layers during the coating process. In the initial step, the first layer of CS on FP was deposited through electrostatic interaction between the positively charged CS at an acidic pH of 4.0 and the negative charge arising from a large number of hydroxyl groups presenting on the cellulose fiber. After coating, the CS/FP possesses a positive charge due to the amine-rich functional groups of CS under the assembled condition at pH 4.0, which is lower than its p*K*_a_ of 6.5. This positively charged surface facilitated the second layer coating of the negatively charged ALG containing numerous carboxyl groups at the assembled pH of 9.0. Consequently, the bilayer of ALG/CS on FP was successfully formed through electrostatic interactions as evident in the significant increase of the ALG/CS/FP thickness in Figure S1. Following the deposition of biopolymers, the plasmonic papers were fabricated *via* the *in situ* growth of AuNPs on the ALG/CS/FP. The coated FPs were immersed into a 0.44 mM gold chloride solution, followed by a heating and drying process. To characterize the *in situ* synthesis of AuNPs and the plasmonic paper preparation process, EDS analysis was conducted in each step of plasmonic paper fabrication, and the results are shown in Figure S2. The percentages weights of four elements for FP, CS/FP, ALG/CS/FP, and AuNP-ALG/CS/FP are shown in Figure S2e, consisting of carbon (C), oxygen (O), and nitrogen (N), which are the main components of the filter paper, and polymers, as well as gold (Au) from the nanoparticles. The presence of N confirms the polymer coating, while the successful formation of AuNPs is clearly affirmed by the high percentage weight of Au. In this respect, CS and ALG on the FP can reduce the precursor Au(III) to Au(0) *via* charge transfer between the empty d-orbitals in Au atoms and the lone-pair electrons from the N or O atoms of amino, carboxyl, and hydroxyl groups of CS and ALG to form AuNPs as described previously.^30,31^ Additionally, AuNP formation on the monolayer coatings, CS/FP and ALG/FP, was demonstrated to compare and realize the effect of double-layered polymer embedding.

**Figure 1:**
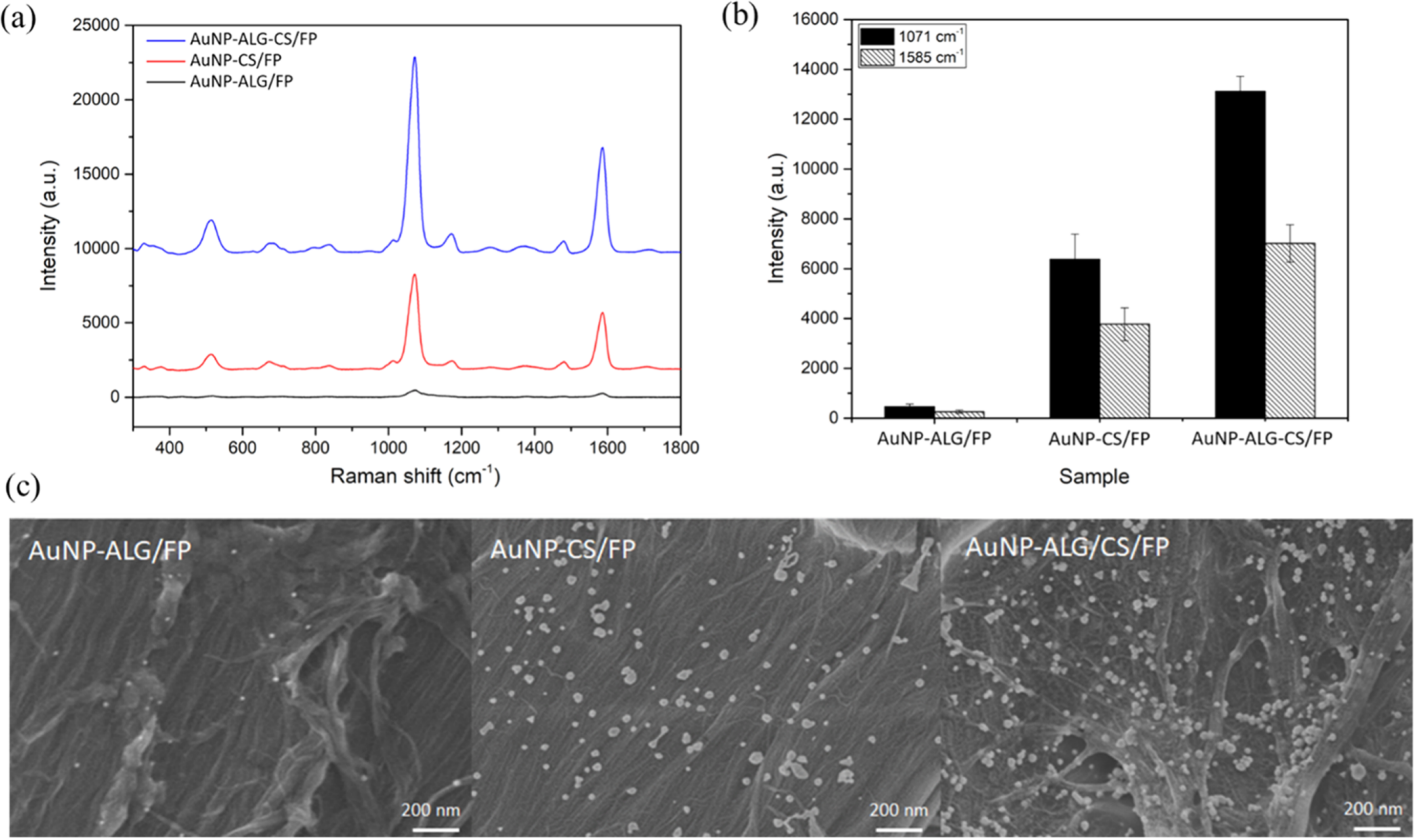
Effect of the LbL assembly of biopolymers on the plasmonic papers: (a) SERS spectra of 10^–4^ M 4-MBA obtained on the plasmonic papers, (b) the intensity of the SERS peaks collected from the plasmonic papers at 1071 cm^–1^ (filled bar) and 1585 (striped bar) cm^–1^, and (c) SEM images of AuNP-ALG/FP, AuNP-CS/FP, and AuNP-ALG/CS/FP, respectively.

After fabrication, the SERS performance of the AuNP-ALG/CS/FP substrate was evaluated in comparison to the AuNP-CS/FP and AuNP-ALG/FP substrates using a Raman probe, 4-MBA. A benchtop Raman spectrometer, equipped with a 785 nm excitation light source, was used to collect SERS spectra of 4-MBA from three distinct substrates, AuNP-ALG/FP, AuNP-CS/FP, and AuNP-ALG/CS/FP as illustrated in Figure 1a. The SERS spectra in Figure 1a showed two characteristic peaks of 4-MBA at 1071 and 1585 cm^–1^, corresponding to the υ(C–C) ring breathing and stretching modes, respectively.^32^ The SERS intensities of two dominant Raman shifts of 4-MBA achieved from the plasmonic papers are plotted in Figure 1b. It was observed that SERS intensities of 4-MBA obtained from the plasmonic paper prepared by LbL polymer deposition (AuNP-ALG/CS/FP) were higher than those of the single-layered coatings, AuNP-ALG/FP and AuNP-CS/FP, by approximately 26 times and 2.2 times, respectively. The SEM measurement was carried out to characterize the surface morphology of the plasmonic papers, and the SEM micrographs are presented in Figure 1c. The SEM images revealed the formation of well-distributed AuNPs with the size range of 20–40 nm throughout the rough fiber structure of the papers with minimal aggregation. The size distribution of AuNPs on the AuNP-ALG/CS/FP substrate was determined by a size measurement of 500 nanoparticles. The result revealed the average size of 34.38 ± 5.61 nm, and the size distribution diagram is depicted in Figure S3. In addition, the density of AuNPs on each plasmonic paper was determined by counting the nanoparticles in various areas by using ImageJ analysis. The result indicated that the plasmonic paper derived from a bilayer deposition, AuNP-ALG/CS/FP, exhibited the highest density of AuNPs of 94 ± 7.8 particles per μm^2^, followed by AuNP-CS/FP and AuNP-ALG/FP (39 ± 7.6 and 10 ± 3.4 particles per μm^2^), respectively. In the case of the monolayer ALG/FP substrate, we hypothesized that Au cations primarily bind to the carboxyl groups of ALG *via* the metal-ion exchange, subsequently reducing to Au(0) facilitating the formation of AuNPs.^33^ Conversely, CS demonstrates superb molecular trapping ability due to its reversible stretching and contracting upon solvent treatment and evaporation. This unique property enables CS to effectively trap Au(III) ions, leading to a high amount of AuNP formation inside the CS layer.^34^ Therefore, the number of AuNPs on CS/FP was notably greater than that on the plasmonic paper derived from the *in situ* reduction of ALG/FP. In the case of the double-layered coating, a notable increase in the number of AuNPs formed on the substrate was evident in the SEM image displayed in Figure 1c. This observation can be explained through the concept of *in situ* synthesis of nanoparticles within polyelectrolyte multilayers (PEMs), as previously reported.^33,35^ In the formation of PEMs, the multilayer assembly is driven by electrostatic interaction between oppositely charged polyelectrolytes or polymers deposited onto substrates. These densely packed multilayer polymers not only create a high density of nucleation sites but also offer spatially controlled regions for nanoparticle formation. Consequently, this polymer multilayer film serves as an effective nanoreactor for the *in situ* synthesis of numerous nanoparticles with well-controlled size distribution and minimal aggregation. In line with our study, CS and ALG, possessing opposite charges, can form a compact bilayer through electrostatic interactions, effectively acting as a nanoreactor for the *in situ* growth of AuNPs. Furthermore, the synergetic effect between CS and ALG enhances the capacity for ion trapping, followed by metal-ion exchange within the bilayer structure. As a result, Au(III) ions can tightly bind to the ALG layer and become trapped in the CS layer of ALG/CS/FP upon immersion and then undergo reduction to yield a high amount of zerovalent AuNPs.

**Figure 2:**
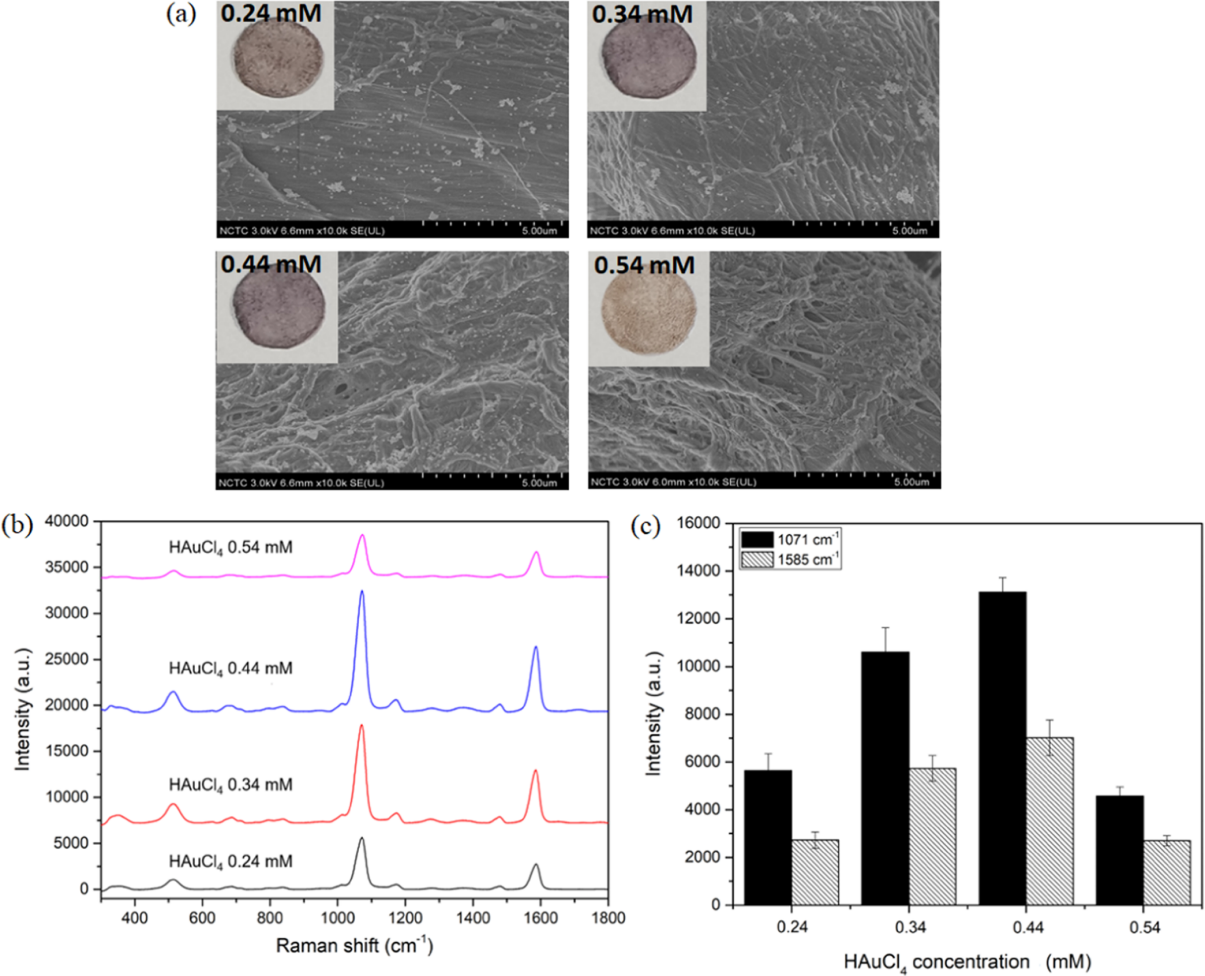
(a) Photographs and SEM images taken at a magnification of 10000x showing the formation of AuNPs on ALG/CS/FP with various HAuCl_4_ concentrations (0.24, 0.34, 0.44, and 0.54 mM), (b) SERS spectra, and (c) related SERS intensity of 10^–4^ M 4-MBA at 1071 (filled bar) and 1585 (striped bar) cm^–1^ achieved from the plasmonic papers.

A high density of AuNPs on the plasmonic paper is expected to enhance the SERS performance by generating a large number of hotspots. To investigate this hypothesis, the influence of gold chloride concentration on AuNP formation and the resulting signal enhancement on the substrate was studied. Various concentrations of gold chloride ranging from 0.24 to 0.54 mM were utilized for the plasmonic paper preparation, consistent with the previous experiments. The SEM images and photographs of the resultant plasmonic papers are shown in Figure 2a. It was observed that increasing the concentration of gold chloride improved AuNP formation and intensified the color of the plasmonic paper. Moreover, the color of the plasmonic paper changed to a purplish blending with a brown–gold color when the gold chloride concentration reached 0.54 mM, indicating the formation of AuNP clumps. The Raman probe, 4-MBA, was applied to test the SERS response of the fabricated AuNP-ALG/CS/FP substrate, and the results are depicted in Figure 2b,c. The signal intensity correlated with the amount of AuNP growth on the plasmonic paper due to the increase in the HAuCl_4_ concentration. The increased density of AuNPs and reduced interparticle spacing in AuNP-ALG/CS/FP substrates promote the coupling of localized surface plasmon resonances (LSPRs), which arises from the collective oscillation of electrons of metallic nanoparticles. This led to the creation of numerous electromagnetic hotspots, particularly at the gaps among AuNPs, thereby amplifying the SERS signal. The highest SERS intensity of 4-MBA was observed on the SERS substrate prepared using 0.44 mM gold chloride solution. However, the SERS intensity of 4-MBA reduced when 0.54 mM gold chloride concentration was used due to the formation of bulk gold on the substrate, suppressing surface plasmon resonance property. Furthermore, random bulk gold formation can cause low spot-to-spot reproducibility of the SERS signal within the same substrate. Consequently, the plasmonic paper prepared with an LbL coating of ALG/CS/FP with 0.44 mM HAuCl_4_ was identified as the optimal condition for fabricating the SERS substrate.

### 2.2 Performance of the Plasmonic Paper

**Figure 3:**
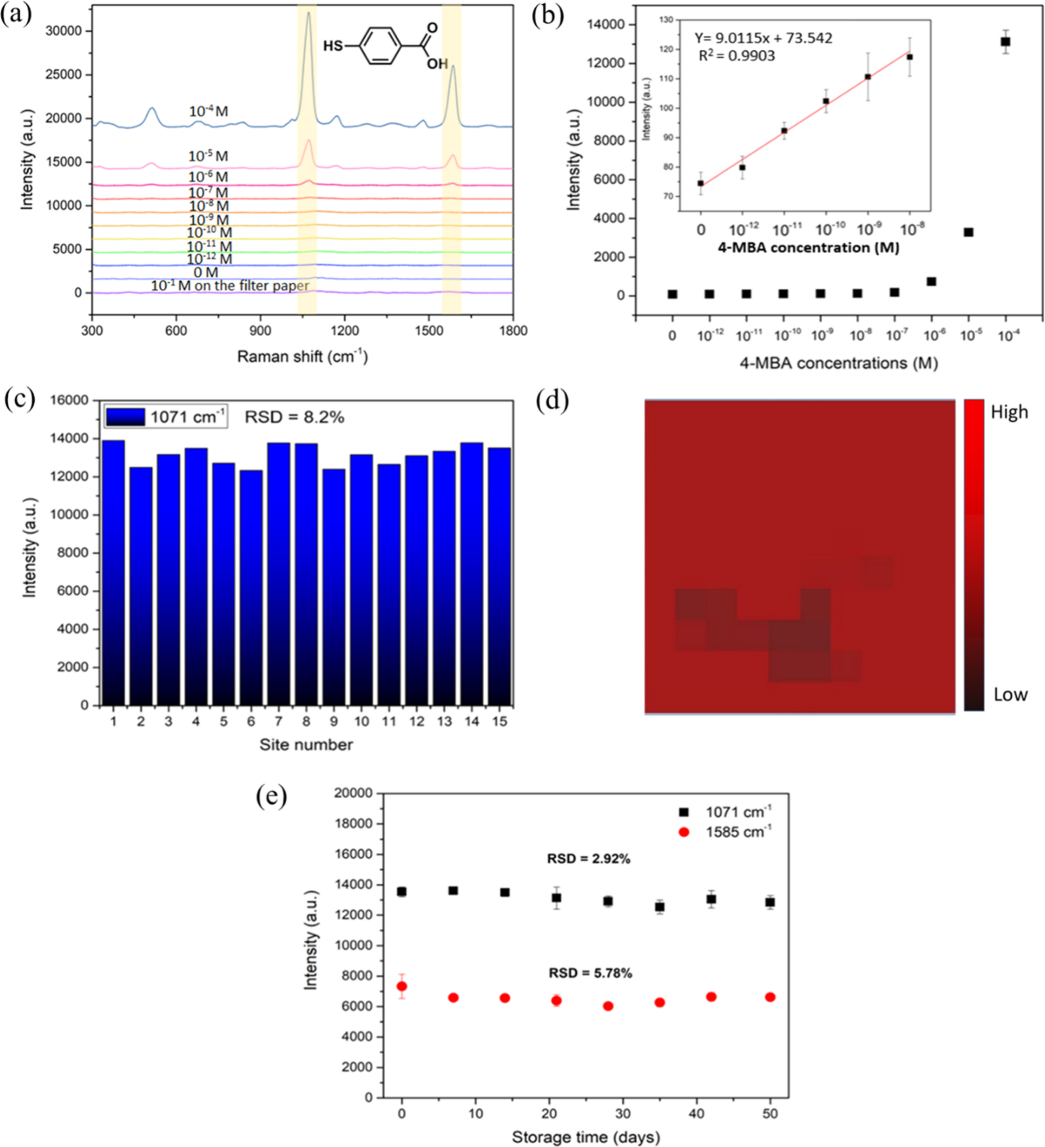
(a) SERS spectra of various concentrations of 4-MBA. (b) A plot between SERS intensities at 1071 cm^–1^ and 4-MBA with various concentrations. (c) Histogram of SERS intensity at the Raman shift of 1071 cm^–1^ from 15 random positions on the plasmonic paper. (d) SERS mapping images at 1071 cm^–1^, which is a characteristic Raman shift of 4-MBA. (e) Average SERS intensities of 1 mM 4-MBA on the substrate at 1071 and 1585 cm^–1^ with various storage times.

Herein, the performance of the fabricated plasmonic paper as a SERS substrate was evaluated using various concentrations of 4-MBA in the range of 10^–12^ to 10^–4^ M. Figure 3a illustrates Raman and SERS spectra of bare plasmonic paper, 4-MBA on FP, and 4-MBA on plasmonic papers. The magnified SERS spectra at low concentrations of 4-MBA are shown in Figure S4. Notably, no background signal that could interfere with the target detection was observed on the bare plasmonic paper. The results showed that the SERS intensity derived from the plasmonic paper increased with the concentration of 4-MBA. The averaged SERS intensities of 4-MBA at a Raman shift of 1071 cm^–1^ were plotted against their correlated concentrations, as depicted in Figure 3b. The linear regression equation was found to be *y* = 9.0115*x* + 73.542 with a correlation coefficient (*R*^2^) of 0.9903. The limit of detection (LOD) was calculated according to the formula LOD = 3σ/*S*, where *S* is the slope of the linear regression equation and σ is the standard deviation of the blank value. Remarkably, the LOD of 4-MBA was exceptionally low as 1.37 × 10^–12^ M, whereas that on the filer paper was about 10^–1^ M.

The signal enhancement in terms of EF was then determined and calculated based on the SERS intensity at 1071 cm^–1^, which was the most intense characteristic Raman shift of 4-MBA, using the following equation
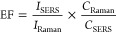
where *I*_SERS_ and *I*_Raman_ are SERS and Raman intensity of 4-MBA at 1071 cm^–1^ band on the plasmonic paper and bare filter paper, respectively. *C*_SERS_ and *C*_Raman_ are the related concentrations of 4-MBA on the plasmonic paper and bare filter paper, correspondingly. According to the calculation in the Supporting Information (Table S1), the EF of the plasmonic paper could be calculated to be 5.7 × 10^10^, representing excellent SERS property.

### 2.3 Reproducibility and Stability of the Plasmonic Paper

To determine the signal uniformity of the plasmonic paper, SERS spectra of 10^–4^ M 4-MBA were acquired with different 15 random spots, and the band intensities at 1071 cm^–1^ are shown in Figure 3c. The relative standard deviation (RSD) of SERS intensities at 1071 cm^–1^ was calculated to be 8.2%, representing a good reproducible SERS substrate. Additionally, to validate the signal uniformity, a SERS mapping experiment was performed and analyzed. The mapping image was carried out with a step size of 1 μm, covering the area of 10 × 10 μm^2^ of the substrate treated by 10^–4^ M 4-MBA. The distinctive Raman shift of 4-MBA at 1071 cm^–1^ was selected and displayed by color coding. The bright red color bar represented high SERS intensities, while the dark color indicated lower signal levels. As seen in Figure 3d, a majority of the areas in the mapping image exhibited a bright red color, indicating high SERS intensity. Conversely, only 10–12% of the images displayed dark colors, representing areas of a low signal. This outcome refers to the high signal uniformity of the substrate, corresponding to the low RSD percentage of the SERS signal derived from the measurement of 15 random spots.

The high signal uniformity observed was a result from homogeneous coating of ALG/CS on the FP, enabling uniform growth of AuNPs on the substrate. In addition to signal uniformity, assessing the stability of the SERS substrate is crucial for practical applications. To evaluate the shelf life of the plasmonic paper, the papers were stored in sealed bags containing nitrogen gas at room temperature for a period of 50 days. Subsequently, SERS spectra of 10^–4^ M 4-MBA on the substrates were collected at different time points, as shown in Figure 3e. It has been found that the SERS intensities of 4-MBA remained considerably stable throughout the storage period, with the RSD of 2.92 and 5.78% for the Raman shifts at 1071 and 1585 cm^–1^, respectively. This outcome suggests that the prepared plasmonic papers exhibit long-term stability that can be manufactured and stored for at least 50 days while maintaining their SERS performance.

### 2.4 Detection of Food Adulteration

**Figure 4:**
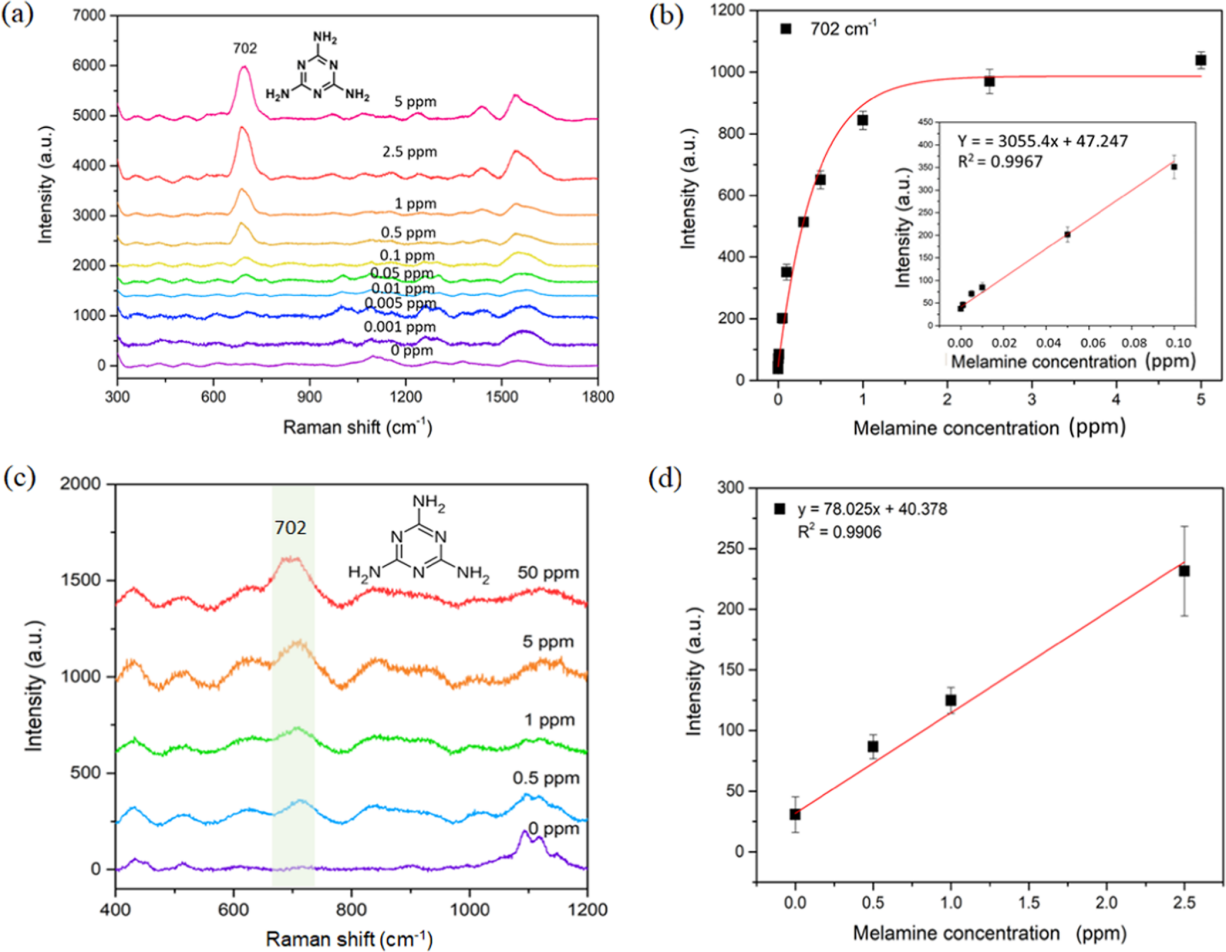
(a) SERS spectra of MEL (0–5 ppm) in solution and (b) the plot of SERS intensity at 702 cm^–1^ corresponding to the concentration. (c) SERS spectra of MEL in milk and (d) the concentration-dependent SERS intensity at 702 cm^–1^ on the plasmonic paper.

The effective onsite identification of contaminated substances in food or the environment is a significant advantage of SERS analysis. MEL is a nitrogen-rich compound containing 66% nitrogen by mass, which is illegally added into dairy-related food products as a means of increasing the total protein content. Ingesting MEL can result in renal failure and can react with some intermediate degradation products, *e.g*., cyanuric acid, resulting in formation of insoluble crystals that lead to kidney stones.^36^ In this study, the detection of MEL was demonstrated by simply immersing the plasmonic paper into various concentrations of MEL solutions, followed by SERS measurements, as previously explained in the Experimental Section. Figure 4a,b displays the SERS spectra of MEL in solution and the plot between SERS intensities and their corresponding concentrations. The dominant peaks of MEL at 702 cm^–1^ were clearly observed in the SERS spectra, which assigned to the ring-breathing II mode involving the in-plane deformation of the triazine ring.^37,38^ It was observed that the SERS intensity increased with MEL concentrations. A linear relationship between MEL concentrations and their corresponding SERS intensities was found in the range of 0–0.1 ppm, expressed by the equation *y* = 3055.4*x* + 47.247 with *R*^2^ = 0.9967. Moreover, the low LOD at 0.2 ppb indicated the high sensitivity and excellent enhancement properties of the fabricated plasmonic paper. This suggests its potential applicability in real sample analysis for detecting trace amounts of MEL.

To demonstrate its practical application in food analysis, plasmonic paper was utilized to detect MEL in spiked milk samples. It is worth noting that detecting MEL in milk can interfere with various components, including proteins, carbohydrates, and fats. Thus, these components were extracted from liquid milk by adding trichloroacetic acid for protein precipitation, followed by centrifugation and filtration, as described previously. Different concentrations of spiked MEL (0, 0.5, 1, 2.5, 5, and 50 ppm) in milk samples were then analyzed on the plasmonic papers after the protein extraction process, following the protocol used earlier. As shown in Figure 4c, the characteristic peak of MEL at 702 cm^–1^ was clearly observed and increased with the concentration of MEL. Importantly, the signal was still observed when the MEL concentration was reduced to 0.5 ppm, which was sufficient for the maximal residue limit (MRL) of MEL in food according to World Health Organization (WHO) and the United States Food and Drug Administration USFDA regulations (2.5 ppm).^39,40^ The LOD of MEL was calculated to be 0.44 ppm using the linear equation *y* = 78.025*x* + 40.378 and the *R*^2^ of 0.9906, as shown in Figure 4d. A comparison of MEL detection in milk samples using the SERS technique on different SERS substrates is depicted in Table S2. It is noteworthy that while some previous studies have achieved LODs at the ppb or ppt level, the fabrication process for those substrates was complex and required additional instruments. Our plasmonic paper, which could be easily prepared by using laboratory equipment and filter paper, showed advantages, such as simplicity, efficiency, and cost-effectiveness for real sample analysis. Moreover, the use of double-layered biopolymers for *in situ* synthesis of AuNPs on the plasmonic paper has never been reported before to the best of our knowledge. To verify the accuracy and reliability of the MEL quantitative determination, a recovery study was performed. Known concentrations of MEL, 0.5, 1, and 2.5 ppm, were spiked in the milk samples, followed by SERS measurement on the plasmonic paper. The detected concentration, percent recovery, and RSD are presented in Table S3. The average recovery of MEL from milk samples was acceptable in the range of 94–103% with RSD values of 3.98–9.00% (*n* = 3). This result confirmed that the proposed SERS substrate is reliable and feasible to detect contaminants in real food samples.

### 2.5 Multiplex Food Adulterations Analysis

**Figure 5:**
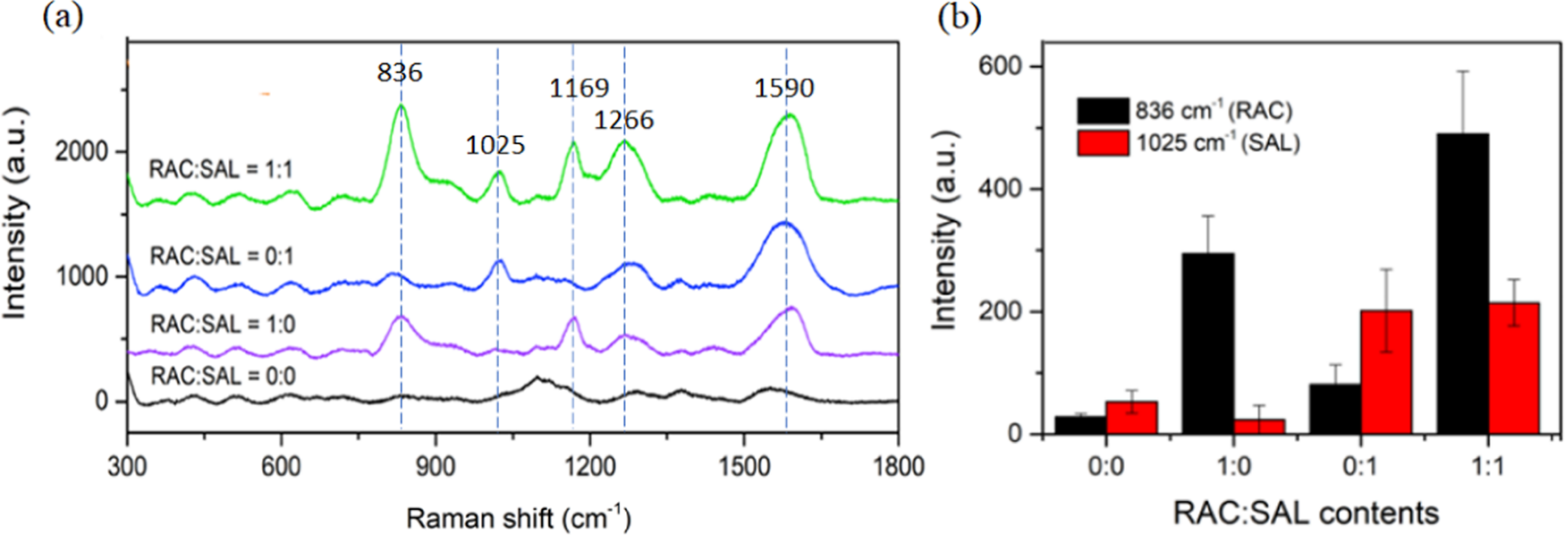
(a) SERS spectra and (b) SERS intensity of multiplex detection of RAC and SAL.

To broaden applications of the fabricated plasmonic paper in food analysis, multiplex detection of β-agonists was demonstrated. RAC and SAL are typical β-agonists, which are misused in animal feeding to increase muscle mass and reduce fat composition in meat.^41^ However, the excessive residues of RAC and SAL can cause serious health problems in human, such as cardiovascular, nausea, and nervousness.^42^ Therefore, RAC and SAL are prohibited as food additives in most countries in the world. In this investigation, we conducted SERS spectra analysis of RAC, SAL, and a mixture of RAC and SAL. Figure S5 shows individual SERS fingerprints of RAC and SAL, along with their structures. The dominant peaks of RAC were observed at 1590, 1266, 1169, and 836 cm^–1^, relating to C=C aromatic stretching, C–H aromatic in-plane stretching coupled with the anilinic C–N stretching and O–H bending, C–N stretching, and C–H aromatic out-of-plane bending, respectively.^43^ SAL exhibited Raman shifts at 1590 cm^–1^ (C=C aromatic stretching), 1025 cm^–1^ ((CH_3_) vibrational mode), and 1266 cm^–1^ (C–H aromatic in-plane stretching vibration coupled with the anilinic C–N stretching and O–H bending). Figure 5a shows the SERS spectra of mixed sample with a specific ratio, including 0:0, 1:0, 0:1, and 1:1 RAC/SAL. In the presence of both RAC and SAL, the characteristic peaks of both compounds were observed at the Raman shifts of 836, 1025, 1169, 1266, and 1590 cm^–1^, as depicted in the green spectrum of Figure 5a. Additionally, the intensities of the characteristic peaks of RAC at 836 cm^–1^ and SAL at 1025 cm^–1^ are plotted in Figure 5b. It is interesting to note that RAC tended to exhibit a strong Raman shift at 836 cm^–1^, while SAL showed not only a high SERS intensity at 1025 cm^–1^ but also a low signal at 836 cm^–1^. Consequently, the mixture containing both RAC and SAL exhibited a SERS intensity at 836 cm^–1^ higher than that of the pure RAC sample. This might be attributed to the signal overlapping of RAC and SAL at the same Raman shift of 836 cm^–1^. On the other hand, the SERS intensities of SAL at 1025 cm^–1^ in both pure sample and the mixture were not considerably different. However, all characteristic Raman shifts of RAC and SAL were preserved in the mixture. This result demonstrated the potential of plasmonic paper as a tool for multiplex determination of real food samples that might contain several contaminants. This capability is crucial in food safety analysis, enabling the detection of multiple contaminants simultaneously, enhancing the efficiency and accuracy of the analysis.

## 3 Conclusions

In summary, this study demonstrated the fabrication of a simple and cost-effective plasmonic paper by the *in situ* synthesis of AuNPs on a filter paper. Biopolymers, CS and ALG, were predeposited on the paper through an LBL assembly as green reducing agents for AuNP formation. The presence of two biopolymers, CS and ALG, significantly enhanced the reduction ability and greatly increased the number of AuNP formation on the substrate. Consequently, signal enhancement was improved due to plasmon coupling of high density of AuNPs on the substrate. The plasmonic paper provides an excellent enhancement factor (EF) of 5.7 × 10^10^ with high sensitivity in the picomolar range for 4-MBA detection. The substrate exhibited good spot-to-spot reproducibility and retained high stability after 50 days of storage. The paper-based SERS substrate was successfully applied for the quantitative detection of MEL in milk with excellent sensitivity at the parts per billion level, which was below the maximal residue limit (MRL). Furthermore, the multiplex detection of 2 β-agonists, RAC and SAL, was demonstrated to exhibit potential applications in food contaminant analysis. In conclusion, this straightforward, paper-based SERS substrate holds promise for applications in food chemistry. Its green *in situ* synthesis approach aligns with environmentally friendly practices, making it a versatile tool in various fields.

## 4 Experimental Section

### 4.1 Chemicals and Reagents

High-molecular-weight chitosan (CS, molecular weight of 310–375 kDa with ≥75% deacetylation), sodium alginate (ALG), gold(III) chloride trihydrate (HAuCl_4_·3H_2_O), 4-mercaptobenzoic acid (4-MBA), melamine (MEL), salbutamol (SAL), and ractopamine hydrochloride (RAC) were purchased from Sigma-Aldrich. Glacial acetic acid was purchased from Carlo Erba. All of the reagents were used as received without further purification. Deionized water was used throughout the experiments and the rinsing process. The cellulose filter paper Whatman no. 1 with a 0.18 mm thickness was supplied from GE Healthcare Life Sciences.

### 4.2 Preparation of ALG- and CS-Coated FP (ALG/CS/FP) by Layer-by-Layer Assembly

FP was cut into a circle shape with a diameter of 6 mm by a paper hole puncher and immersed into CS (1 wt % in 1% acetic acid) for 1 h. After that, the CS/FP was rinsed with deionized water to remove unbound CS and dipped into 1 wt % ALG solution in water at pH 9.0 after adjusting by 0.1 M NaOH for 5 min. Then, the double-coated paper (ALG/CS/FP) was rinsed with deionized water and dried at room temperature.

### 4.3 Fabrication of the Plasmonic Paper AuNP-ALG/CS/FP

All glassware was prior cleaned by aqua regia solution (HCl/HNO_3_, 3:1) and then rinsed with deionized water. AuNPs were synthesized on the coated FP through *in situ* reduction of gold chloride by the ALG/CS. First, 5 mL of gold chloride solution with various concentrations was added into Duran bottles, followed by the addition of ALG/CS/FP, and the reaction temperature was set at 80 °C. The reaction was stirred at 50 rpm and proceeded for 30 min. After that, the prepared plasmonic papers were washed with DI water and dried in an oven at 37 °C. The formation and distribution of AuNPs on the resulting plasmonic paper were observed by scanning electron microscopy (SEM) using an FE-SEM SU8030 (Hitachi, Japan) with an acceleration voltage of 3.0 kV and an electron current of 5 μA.

### 4.4 SERS Analysis

SERS and Raman spectra were determined by using a Raman spectrometer (HORIBA scientific benchtop Raman spectrometer) with a laser at a wavelength of 785 nm and a power of 29.8 mW. The system was calibrated by measuring the Raman signal acquired from a standard silicon wafer at a wavenumber of 520 cm^–1^. SERS and Raman spectra were recorded in the range of 300 to 1800 cm^–1^. To determine SERS performance, the plasmonic paper was dipped in 200 μL of a 4-MBA solution in DMSO with various concentrations for 30 min and dried at room temperature for 30 min before SERS analysis. SERS spectra were recorded on 3 samples and 15 random positions on each sample with an acquisition time of 10 s and 3 accumulations. After SERS spectra were acquired, the sensitivity and EF of the plasmonic paper were calculated. The Raman images were obtained by using a Raman point-mapping method. SERS mapping images were collected with a 1.0 μm step size over the specific area of 10 × 10 μm^2^ with an integration time of 1 s.

### 4.5 Determination of Food Additives

Ten milligrams of MEL was dissolved in 10 mL of the mixture between methanol and ethanol with a ratio of 1:1 to achieve 1000 ppm of stock solution. To demonstrate the feasibility of real sample analysis, MEL solution was added to milk and diluted to achieve various concentrations. One percent trichloroacetic acid was added to the spiked milk samples with a ratio of 1:1 (spiked milk sample/acid) to precipitate milk proteins. The mixture was then vortexed and sonicated for 5 min, followed by centrifugation for 10 min at 12,000 rpm. The supernatant was collected and filtered with a 0.22 μm PVDF membrane. The pH of the supernatant was then adjusted to 7.0 by 0.1 M NaOH. The plasmonic paper was then immersed in 200 μL of the spiked sample for 30 min and dried before SERS analysis. The characteristic peak of MEL at 702 cm^–1^ was determined while the nonspiked sample was used as a control. For the multiplex detection model, 2 β-agonists, RAC and SAL, were mixed to achieve different ratios, including 1:0, 0:1, and 1:1 RAC/SAL. SERS spectra of both individual and mixture samples were observed, and SERS intensities of their characteristic Raman shifts were plotted at 836 and 1025 cm^–1^ for RAC and SAL, respectively.

## Supporting Information Available

Cross-sectional SEM images of the bare and pretreated paper; EDS analysis of each step of plasmonic paper fabrication; size distribution of AuNPs on the plasmonic paper; SERS spectra of 4-MBA on the plasmonic papers; SERS fingerprints of RAC and SAL; enhancement factor table of the plasmonic paper; comparison table of the detection of melamine in milk; sample; and recovery study table of melamine spiked in milk (PDF)

## References

[ref1] Ellis D. I.; Brewster V. L.; Dunn W. B.; Allwood J. W.; Golovanov A. P.; Goodacre R. Fingerprinting food: current technologies for the detection of food adulteration and contamination. Chem. Soc. Rev. 2012, 41, 5706–5727. 10.1039/c2cs35138b. 22729179

[ref2] Cordella C.; Moussa I.; Martel A. C.; Sbirrazzuoli N.; Lizzani-Cuvelier L. Recent Developments in Food Characterization and Adulteration Detection: Technique-Oriented Perspectives. J. Agric. Food Chem. 2002, 50, 1751–1764. 10.1021/jf011096z. 11902909

[ref3] Zhang D.; Pu H.; Huang L.; Sun D. W. Advances in flexible surface-enhanced Raman scattering (SERS) substrates for nondestructive food detection: Fundamentals and recent applications. Trends Food Sci. Technol. 2021, 109, 690–701. 10.1016/j.tifs.2021.01.058.

[ref4] Perumal J.; Wang Y.; Attia A. B. E.; Dinish U. S.; Olivo M. Towards a point-of-care SERS sensor for biomedical and agri-food analysis applications: a review of recent advancements. Nanoscale 2021, 13, 553–580. 10.1039/D0NR06832B. 33404579

[ref5] Haes A. J.; Zou S.; Zhao J.; Schatz G. C.; Van Duyne R. P. Localized Surface Plasmon Resonance Spectroscopy near Molecular Resonances. J. Am. Chem. Soc. 2006, 128, 10905–10914. 10.1021/ja063575q. 16910686

[ref6] Pilot R.; Signorini R.; Durante C.; Orian L.; Bhamidipati M.; Fabris L. A Review on Surface-Enhanced Raman Scattering. Biosensors 2019, 9, 57 10.3390/bios9020057. 30999661 PMC6627380

[ref7] Uskoković-Marković S.; Kuntić V.; Bajuk-Bogdanović D.; Holclajtner-Antunović I. Surface-Enhanced Raman Scattering (SERS) Biochemical Applications. In Encyclopedia of Spectroscopy and Spectrometry, 3rd ed.; Lindon J. C.; Tranter G. E.; Koppenaal D. W., Eds.; Academic Press: Oxford, 2017; pp 383–388.

[ref8] Sharma B.; Frontiera R. R.; Henry A.-I.; Ringe E.; Van Duyne R. P. SERS: Materials, applications, and the future. Mater. Today 2012, 15, 16–25. 10.1016/S1369-7021(12)70017-2.

[ref9] Yuen C.; Zheng W.; Huang Z. Surface-enhanced raman scattering: Principles, nanostructures, fabrications, and biomedical application. J. Innov. Opt. Health Sci. 2008, 1, 267–284. 10.1142/s179354580800025x.

[ref10] Arabi M.; Ostovan A.; Zhang Z.; Wang Y.; Mei R.; Fu L.; Wang X.; Ma J.; Chen L. Label-free SERS detection of Raman-Inactive protein biomarkers by Raman reporter indicator: Toward ultrasensitivity and universality. Biosens. Bioelectron. 2021, 174, 112825 10.1016/j.bios.2020.112825. 33243696

[ref11] Villa J. E. L.; Afonso M. A. S.; Dos Santos D. P.; Mercadal P. A.; Coronado E. A.; Poppi R. J. Colloidal gold clusters formation and chemometrics for direct SERS determination of bioanalytes in complex media. Spectrochim. Acta, Part A 2020, 224, 117380 10.1016/j.saa.2019.117380. 31344581

[ref12] Halvorson R. A.; Vikesland P. J. Surface-Enhanced Raman Spectroscopy (SERS) for Environmental Analyses. Environ. Sci. Technol. 2010, 44, 7749–7755. 10.1021/es101228z. 20836559

[ref13] Azoulay J.; Débarre A.; Richard A.; Tchénio P.; Bandow S.; Iijima S. Polarised Raman spectroscopy on a single class of single-wall nanotubes by nano surface-enhanced scattering. Chem. Phys. Lett. 2000, 331, 347–353. 10.1016/S0009-2614(00)01189-1.

[ref14] Ma X.; Wen S.; Xue X.; Guo Y.; Jin J.; Song W.; Zhao B. Controllable Synthesis of SERS-Active Magnetic Metal–Organic Framework-Based Nanocatalysts and Their Application in Photoinduced Enhanced Catalytic Oxidation. ACS Appl. Mater. Interfaces 2018, 10, 25726–25736. 10.1021/acsami.8b03457. 29987930

[ref15] Hu J.; Wang S.; Wang L.; Li F.; Pingguan-Murphy B.; Lu T. J.; Xu F. Advances in paper-based point-of-care diagnostics. Biosens. Bioelectron. 2014, 54, 585–597. 10.1016/j.bios.2013.10.075. 24333570

[ref16] Yetisen A. K.; Akram M. S.; Lowe C. R. Paper-based microfluidic point-of-care diagnostic devices. Lab Chip 2013, 13, 2210–2251. 10.1039/c3lc50169h. 23652632

[ref17] Credou J.; Berthelot T. Cellulose: from biocompatible to bioactive material. J. Mater. Chem. B 2014, 2, 4767–4788. 10.1039/C4TB00431K. 32261769

[ref18] Jiang Q.; Chandar Y. J.; Cao S.; Kharasch E. D.; Singamaneni S.; Morrissey J. J. Rapid, Point-of-Care, Paper-Based Plasmonic Biosensor for Zika Virus Diagnosis. Adv. Biosyst. 2017, 1, 1700096 10.1002/adbi.201700096. 32646188

[ref19] Shangguan J. W.; Liu Y.; Wang S.; Hou Y. X.; Xu B. Y.; Xu J. J.; Chen H. Y. Paper Capillary Enables Effective Sampling for Microfluidic Paper Analytical Devices. ACS Sens. 2018, 3, 1416–1423. 10.1021/acssensors.8b00335. 29873481

[ref20] Xie J.; Li L.; Khan I. M.; Wang Z.; Ma X. Flexible paper-based SERS substrate strategy for rapid detection of methyl parathion on the surface of fruit. Spectrochim. Acta, Part A 2020, 231, 118104 10.1016/j.saa.2020.118104. 32006913

[ref21] Zhang K.; Ji J.; Fang X.; Yan L.; Liu B. Carbon nanotube/gold nanoparticle composite-coated membrane as a facile plasmon-enhanced interface for sensitive SERS sensing. Analyst 2015, 140, 134–139. 10.1039/C4AN01473A. 25347701

[ref22] Godoy N. V.; García-Lojo D.; Sigoli F. A.; Pérez-Juste J.; Pastoriza-Santos I.; Mazali I. O. Ultrasensitive inkjet-printed based SERS sensor combining a high-performance gold nanosphere ink and hydrophobic paper. Sens. Actuators, B 2020, 320, 128412 10.1016/j.snb.2020.128412.

[ref23] Zhang R.; Xu B.-B.; Liu X.-Q.; Zhang Y.-L.; Xu Y.; Chen Q.-D.; Sun H.-B. Highly efficient SERS test strips. Chem. Commun. 2012, 48, 5913–5915. 10.1039/c2cc31604h. 22572925

[ref24] Cheng M.-L.; Tsai B.-C.; Yang J. Silver nanoparticle-treated filter paper as a highly sensitive surface-enhanced Raman scattering (SERS) substrate for detection of tyrosine in aqueous solution. Anal. Chim. Acta 2011, 708, 89–96. 10.1016/j.aca.2011.10.013. 22093349

[ref25] Kim W.; Kim Y.-H.; Park H.-K.; Choi S. Facile Fabrication of a Silver Nanoparticle Immersed, Surface-Enhanced Raman Scattering Imposed Paper Platform through Successive Ionic Layer Absorption and Reaction for On-Site Bioassays. ACS Appl. Mater. Interfaces 2015, 7, 27910–27917. 10.1021/acsami.5b09982. 26619139

[ref26] Kim W.; Lee J. C.; Shin J. H.; Jin K. H.; Park H. K.; Choi S. Instrument-Free Synthesizable Fabrication of Label-Free Optical Biosensing Paper Strips for the Early Detection of Infectious Keratoconjunctivitides. Anal. Chem. 2016, 88, 5531–5537. 10.1021/acs.analchem.6b01123. 27127842

[ref27] Lee K. X.; Shameli K.; Yew Y. P.; Teow S. Y.; Jahangirian H.; Rafiee-Moghaddam R.; Webster T. J. Recent Developments in the Facile Bio-Synthesis of Gold Nanoparticles (AuNPs) and Their Biomedical Applications. Int. J. Nanomed. 2020, 15, 275–300. 10.2147/IJN.S233789. PMC6970630 32021180

[ref28] Facchi P. D.; da Cruz A. J.; Bonafé G. E.; Pereira G. B. A.; Fajardo R. A.; Venter A. S. S.; Monteiro P. J.; Muniz C. E.; Martins F. A. Polysaccharide-Based Materials Associated with or Coordinated to Gold Nanoparticles: Synthesis and Medical Application. Curr. Med. Chem. 2017, 24, 2701–2735. 10.2174/0929867324666170309123351. 28294043

[ref29] Huang H.; Yang X. Synthesis of Chitosan-Stabilized Gold Nanoparticles in the Absence/Presence of Tripolyphosphate. Biomacromolecules 2004, 5, 2340–2346. 10.1021/bm0497116. 15530050

[ref30] Potara M.; Maniu D.; Astilean S. The synthesis of biocompatible and SERS-active gold nanoparticles using chitosan. Nanotechnol 2009, 20, 315602 10.1088/0957-4484/20/31/315602. 19597258

[ref31] Zhao X.; Li Z.; Deng Y.; Zhao Z.; Li X.; Xia Y. Facile Synthesis of Gold Nanoparticles with Alginate and Its Catalytic Activity for Reduction of 4-Nitrophenol and H_2_O_2_ Detection. Materials 2017, 10, 557 10.3390/ma10050557. 28772911 PMC5459079

[ref32] Orendorff C. J.; Gole A.; Sau T. K.; Murphy C. J. Surface-Enhanced Raman Spectroscopy of Self-Assembled Monolayers: Sandwich Architecture and Nanoparticle Shape Dependence. Anal. Chem. 2005, 77, 3261–3266. 10.1021/ac048176x. 15889917

[ref33] Wang T. C.; Rubner M. F.; Cohen R. E. Polyelectrolyte Multilayer Nanoreactors for Preparing Silver Nanoparticle Composites: Controlling Metal Concentration and Nanoparticle Size. Langmuir 2002, 18, 3370–3375. 10.1021/la015725a.

[ref34] Li D.; Lv D. Y.; Zhu Q. X.; Li H.; Chen H.; Wu M. M.; Chai Y. F.; Lu F. Chromatographic separation and detection of contaminants from whole milk powder using a chitosan-modified silver nanoparticles surface-enhanced Raman scattering device. Food Chem. 2017, 224, 382–389. 10.1016/j.foodchem.2016.12.040. 28159284

[ref35] Logar M.; Jancar B.; Suvorov D.; Kostanjsek R. In situ synthesis of Ag nanoparticles in polyelectrolyte multilayers. Nanotechnology 2007, 18, 325601 10.1088/0957-4484/18/32/325601.

[ref36] Brown C. A.; Jeong K.-S.; Poppenga R. H.; Puschner B.; Miller D. M.; Ellis A. E.; Kang K.-I.; Sum S.; Cistola A. M.; Brown S. A. Outbreaks of Renal Failure Associated with Melamine and Cyanuric Acid in Dogs and Cats in 2004 and 2007. J. Vet. Diagn. Invest. 2007, 19, 525–531. 10.1177/104063870701900510. 17823396

[ref37] Koglin E.; Kip B. J.; Meier R. J. Adsorption and Displacement of Melamine at the Ag/Electrolyte Interface Probed by Surface-Enhanced Raman Microprobe Spectroscopy. J. Phys. Chem. A 1996, 100 (12), 5078–5089. 10.1021/jp953208t.

[ref38] Koglin E.; Kip B. J.; Meier R. J. Adsorption and Displacement of Melamine at the Ag/Electrolyte Interface Probed by Surface-Enhanced Raman Microprobe Spectroscopy. J. Phys. Chem. A 1996, 100, 5078–5089. 10.1021/jp953208t.

[ref39] World Health Organization. Melamine and Cyanuric acid: Toxicity, Preliminary Risk Assessment and Guidance on Levels in Food, 2008.

[ref40] Food and Drug Administration FDA Issues Interim Safety and Risk Assessment of Melamine and Melamine-Related Compounds in Food. https://www.foodingredientsfirst.com/news/fda-issues-interim-safety-and-risk-assessment-of-melamine-and-melamine-related-compounds-in-food.html.

[ref41] Engeseth N. J.; Lee K.-O.; Bergen W. G.; Helferich W. G.; Knudson B. K.; Merkel R. A. Fatty Acid Profiles of Lipid Depots and Cholesterol Concentration in Muscle Tissue of Finishing Pigs Fed Ractopamine. J. Food Sci. 1992, 57, 1060–1062. 10.1111/j.1365-2621.1992.tb11262.x.

[ref42] Brambilla G.; Cenci T.; Franconi F.; Galarini R.; Macrì A.; Rondoni F.; Strozzi M.; Loizzo A. Clinical and pharmacological profile in a clenbuterol epidemic poisoning of contaminated beef meat in Italy. Toxicol. Lett. 2000, 114 (1–3), 47–53. 10.1016/S0378-4274(99)00270-2. 10713468

[ref43] Izquierdo-Lorenzo I.; Sánchez-Cortés S.; García-Ramos J. V. Adsorption of beta-adrenergic agonists used in sport doping on metal nanoparticles: a detection study based on surface-enhanced Raman scattering. Langmuir 2010, 26 (18), 14663–14670. 10.1021/la102590f. 20799745

