# Supplementary material for: Layer-by-Layer Biopolymer Assembly for the In Situ Fabrication of AuNP Plasmonic Paper—A SERS Substrate for Food Adulteration Detection
Source: ACS Omega. 2024 Feb 20;9(9):10099–109. doi: 10.1021/acsomega.3c05966 (PMC10918676; doi:10.1021/acsomega.3c05966)
Supplement: Supplementary file 1 — ao3c05966_si_001.pdf [file ao3c05966_si_001.pdf]

A layer-by-layer biopolymer assembly for the *in-situ* fabrication of AuNPs plasmonic paper - a SERS substrate for food adulteration detection

Nopparat Viriyakitpattana, Chanoknan Rattanabut, Chutiparn Lertvachirapaiboon, Dechnarong Pimalai and Suwussa Bamrungsap*

National Nanotechnology Center, National Science and Technology Development Agency, Thailand Science Park, Phahonyothin Road, Khlong Nueng, Khlong Luang, Pathum Thani, 12120, Thailand

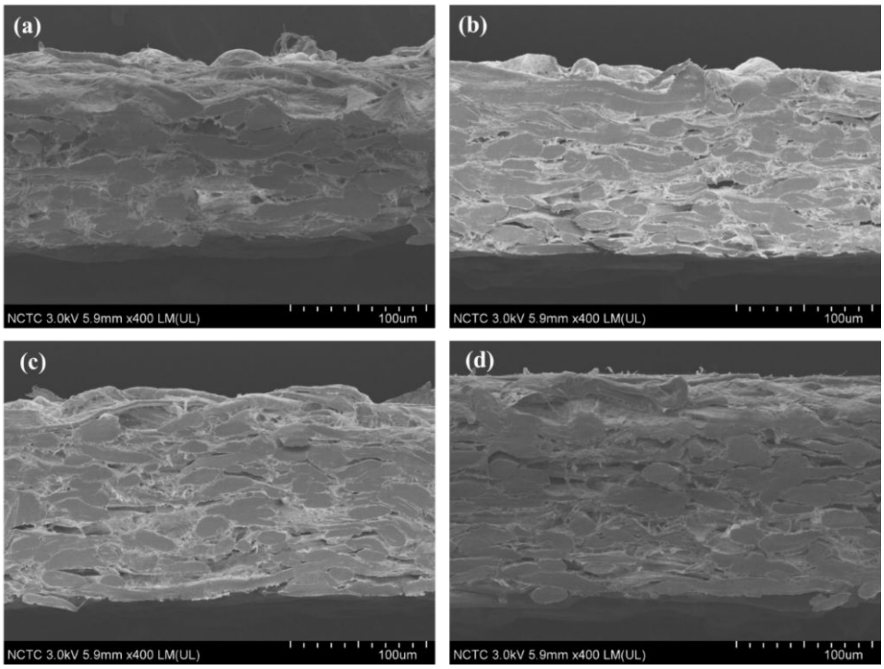

Figure S1 Thickness of the bared and pre-treated paper presented by cross-sectional SEM images of (a) bared filter paper (FP), (b) CS/FP, (c) ALG/FP, and (d) ALG/CS/FP

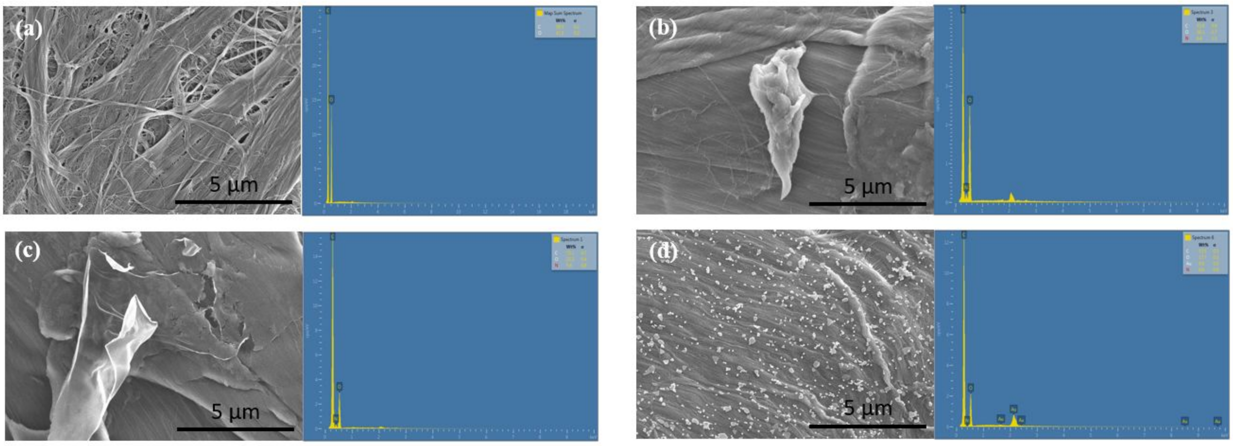

(e)

Samples	C (wt%)	O (wt%)	N (wt%)	Au (wt%)
FP	59.6	40.4	-	-
CS/FP	55.6	38.1	6.4	-
ALG/CS/FP	72.1	22.3	5.6	-
AuNP-ALG/CS/FP	72.5	17.7	-	9.8

Figure S2 EDS analysis of each step of plasmonic paper fabrication (a) FP, (b) CS/FP, (c) ALG/CS/FP, (d) AuNP-ALG/CS/FP, and (e) the percentages of four main elements including C, O, N, and Au, respectively.

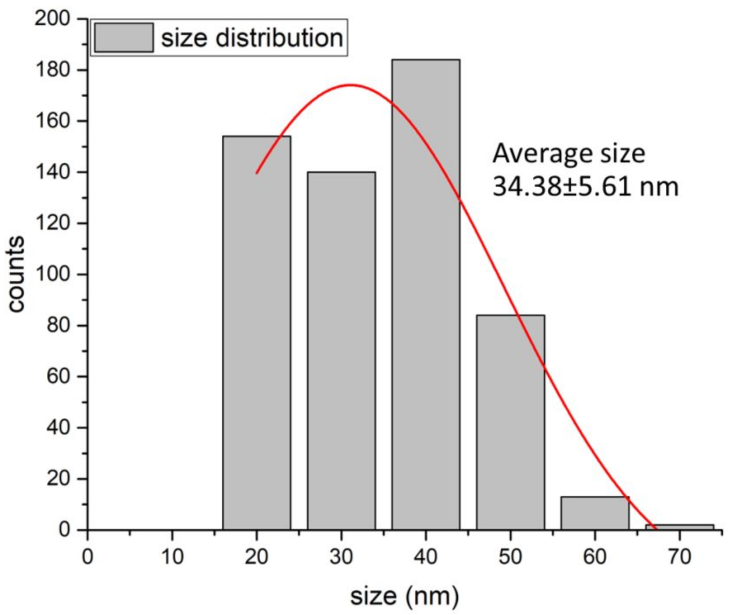

Figure S3 Size distribution of AuNPs on the AuNP-ALG/CS/FP substrate

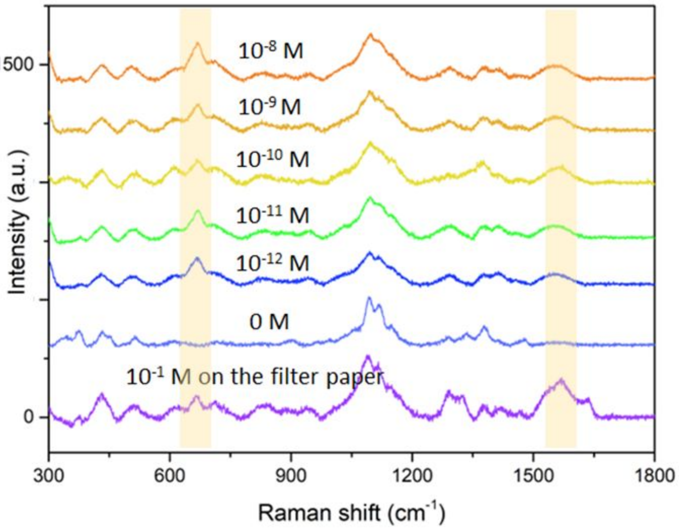

Fig. S4 The magnified SERS spectra of 4-MBA from 0-10^{-8} M on the plasmonic papers

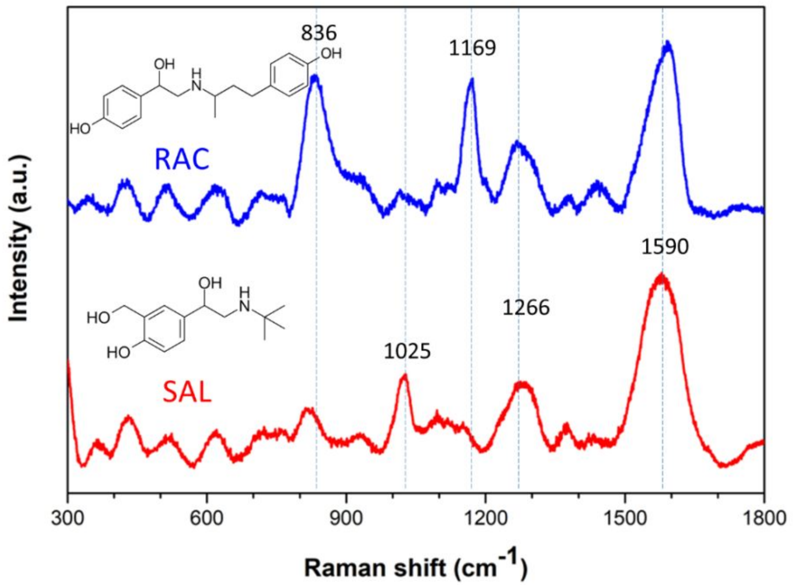

Fig. S5 SERS fingerprints of RAC and SAL including their structures.

Table S1 Enhancement factor of paper-based SERS substrates by 4-mercaptobenzoic acid (4-MBA) compared with filter paper calculated using Raman shift at 1071 cm^{-1}

C_{Raman} (M)	I_{Raman}	C_{SERS} (M)	I_{SERS}	I_{SERS}/I_{Raman}	C_{Raman}/C_{SERS}	EF
10^{-1}	151	10^{-10}	106	0.70198675	10^{9}	7.0×10^{8}
10^{-1}	151	10^{-11}	98	0.64900662	10^{10}	6.5×10^{9}
10^{-1}	151	10^{-12}	86	0.56953642	10^{11}	5.7×10^{10}

Table S2 Comparison of the detection of melamine in milk sample from different substrates with SERS technique

**Samples**	**Limit of detection (ppm)**	**Ref.**
MIP/Ag dendrite	1.51 ppm	(Hu, Feng, Gao, Li-Chan, Grant, & Lu, 2015)
Hollow gold chip	1 ppm	(Guo et al., 2014)
Nanofibrillated cellulose (NFC)/AuNP substrate	1 ppm	(Xiong, Chen, Liou, & Lin, 2017)
AgNP-CS/FP	1 ppm	(D. Li et al., 2017)
Ag nanocube (NC) array substrate	0.5 ppm	(L. Li & Chin, 2021)
Au-capped Si nanopillar SERS substrates	0.3 ppm	(Viehrig et al., 2020)
AgNPs/poly(ε-caprolactone) (PCL) nanofiber membrane	5 ppb	(Shi, You, Gao, Liang, Li, & Yin, 2017)
AgNPs/Au film over nanosphere (AuFON) SERS substrate	0.1 ppb	(Stitt, Wang, Wu, Xiao, Dong, & Wang, 2014)
α-cyclodextrin (CD)-AgNPs	3 ppt	(Ma et al., 2013)
AuNP-ALG/CS/FP	0.44 ppm	This work

Table S3 Recovery study of melamine spiked in milk samples.

Spiked MEL concentration (ppm)	Detected MEL concentration (ppm)	Recovery (%)	RSD (%)
0.5	0.505±0.0224	101±5	4.87
1	1.066±0.0218	103±4	3.98
2.5	2.225±0.0360	94±8	9.00

References

Guo, Z., Cheng, Z., Li, R., Chen, L., Lv, H., Zhao, B., & Choo, J. (2014). One-step detection of melamine in milk by hollow gold chip based on surface-enhanced Raman scattering. *Talanta, 122*, 80-84. https://doi.org/10.1016/j.talanta.2014.01.043.

Hu, Y., Feng, S., Gao, F., Li-Chan, E. C. Y., Grant, E. R., & Lu, X. (2015). Detection of melamine in milk using molecularly imprinted polymers-surface enhanced Raman spectroscopy. *Food Chemistry, 176*, 123-129.

Li, D., Lv, D. Y., Zhu, Q. X., Li, H., Chen, H., Wu, M. M., . . . Lu, F. (2017). Chromatographic separation and detection of contaminants from whole milk powder using a chitosan-modified silver nanoparticles surface-enhanced Raman scattering device. *Food Chemistry, 224*, 382-389. https://doi.org/10.1016/j.foodchem.2016.12.040.

Li, L., & Chin, W. S. (2021). Rapid and sensitive SERS detection of melamine in milk using Ag nanocube array substrate coupled with multivariate analysis. *Food Chemistry, 357*, 129717. https://doi.org/https://doi.org/10.1016/j.foodchem.2021.129717.

Ma, P., Liang, F., Sun, Y., Jin, Y., Chen, Y., Wang, X., . . . Song, D. (2013). Rapid determination of melamine in milk and milk powder by surface-enhanced Raman spectroscopy and using cyclodextrin-decorated silver nanoparticles. *Microchimica Acta, 180*(11-12), 1173-1180. https://doi.org/10.1007/s00604-013-1059-7.

Shi, J., You, T., Gao, Y., Liang, X., Li, C., & Yin, P. (2017). Large-scale preparation of flexible and reusable surface-enhanced Raman scattering platform based on electrospinning AgNPs/PCL nanofiber membrane. *RSC Adv., 7*(75), 47373-47379. https://doi.org/10.1039/c7ra09726c.

Stitt, A., Wang, J. F., Wu, X. Z., Xiao, R., Dong, P. T., & Wang, C. G. (2014). Performance-Enhancing Methods for Au Film over Nanosphere Surface-Enhanced Raman Scattering Substrate and Melamine Detection Application. *PLOS ONE, 9*(6), e97976. https://doi.org/10.1371/journal.pone.0097976.

Viehrig, M., Rajendran, S. T., Sanger, K., Schmidt, M. S., Alstrøm, T. S., Rindzevicius, T., Boisen, A. (2020). Quantitative SERS Assay on a Single Chip Enabled by Electrochemically Assisted Regeneration: A Method for Detection of Melamine in Milk. *Analytical Chemistry, 92*(6), 4317-4325. https://doi.org/10.1021/acs.analchem.9b05060.

Xiong, Z., Chen, X., Liou, P., & Lin, M. (2017). Development of nanofibrillated cellulose coated with gold nanoparticles for measurement of melamine by SERS. *Cellulose, 24*(7), 2801-2811. https://doi.org/10.1007/s10570-017-1297-7.
